# Burden of Rhesus isoimmunization and pregnancy outcomes: a cross-sectional study conducted at Kenyatta National Hospital, Kenya

**DOI:** 10.11604/pamj.2025.52.163.47030

**Published:** 2025-12-17

**Authors:** John Abayo Otieno, Rose Jepchumba Kosgei, Chrisostim Wekesa Barasa, Omondi Ogutu, Rose Betty Mukii

**Affiliations:** 1 Department of Obstetrics and Gynaecology, School of Medicine, University of Nairobi, Nairobi, Kenya,; 2 Monitoring, Evaluation, Research and Learning, Amref Health Africa, Nairobi, Kenya

**Keywords:** Rhesus factor, isoimmunisation, anti-D, pregnancy outcomes

## Abstract

**Introduction:**

the ABO and Rhesus systems remain the most clinically significant blood group antigens on red blood cell membranes. Rhesus isoimmunization occurs when maternal Rh antibodies in a RhD-negative woman react with red blood cells of an RhD-positive fetus, leading to adverse fetal and neonatal outcomes.

**Methods:**

a cross-sectional review of 194 medical records of RhD-negative pregnant women managed at Kenyatta National Hospital (KNH) between 2013 and 2019 was conducted. Data on sociodemographic, obstetric, and clinical characteristics were extracted and analyzed using SPSS version 23. Multivariable logistic regression was performed to determine associations between Rh isoimmunization and adverse pregnancy outcomes. Adjusted odds ratios (aORs) with 95% confidence intervals were calculated, and p<0.05 was considered statistically significant.

**Results:**

the mean age (SD) of participants was 30.1 years, and the mean gestational age at delivery was 38.9 weeks. Most participants were multigravida (69.1%) and married (91.2%). The prevalence of Rh isoimmunization was 4.1%. Isoimmunized women had significantly higher odds of miscarriage (aOR 5.64, 95% CI 1.48-21.53; p=0.01), hydrops fetalis (aOR 8.72, 95% CI 2.10-36.12; p<0.001), intrauterine foetal death (aOR 9.83, 95% CI 2.75-35.12; p<0.001), low birth weight <2500g (aOR 7.40, 95% CI 1.93-28.43; p=0.004), and poor APGAR score <7 at 5 minutes (aOR 10.26, 95% CI 2.98-35.32; p<0.001). Neonates of isoimmunized mothers were also more likely to require neonatal intensive care unit (NICU) admission (aOR 6.92, 95% CI 1.41-33.84; p=0.02).

**Conclusion:**

the prevalence of Rh isoimmunization among RhD-negative women at KNH was 4.1%. Isoimmunization was significantly associated with miscarriage, hydrops fetalis, IUFD, low birth weight, poor APGAR scores, and NICU admission. Strengthening routine anti-D prophylaxis and improving documentation of its administration after pregnancy loss or delivery are critical to reducing isoimmunization and related complications.

## Introduction

The ABO and Rhesus (Rh) systems remain the most clinically significant blood group antigens on red blood cell membranes [1]. The Rh system consists of two related proteins, RhD and RhCE, which express the D and CE antigens, respectively. Individuals with the RhD antigen are Rhesus positive (RhD+), while those without it are Rhesus negative (RhD-) [1]. Globally, the prevalence of RhD-negative blood varies by population, being highest among the Basques of Spain (35%), followed by Caucasians 14% [2], and lowest among sub-Saharan African populations (between 2.4 and 4.5%) [3]. In Kenya, the prevalence has been reported at approximately 3.9% [4]. The relatively low prevalence in African populations [5] does not negate its clinical importance due to the serious consequences of Rhesus isoimmunization, also known as Rh incompatibility [6]. Rhesus iso immunization occurs when an RhD-negative mother is exposed to RhD-positive fetal red blood cells, leading to the production of maternal anti-D antibodies. These antibodies can cross the placenta in subsequent pregnancies and destroy fetal red blood cells, resulting in hemolytic disease of the fetus and newborn. The clinical manifestations range from mild jaundice and anemia to hydrops fetalis, stillbirth, and neonatal death [7,8].

This occurs either from feto-maternal haemorrhage through escape of the foetal cells through the placenta or from incompatible blood transfusion [9]. Predisposing factors for feto-maternal haemorrhage include delivery, spontaneous or induced abortion, ectopic pregnancy, miscarriage, intrauterine foetal death, abdominal trauma, antepartum haemorrhage, amniocentesis, chorionic villous sampling, foetal blood sampling, embryo reduction, shunt insertion, external cephalic version, manual removal of the placenta, and caesarean section [9]. Most sensitization cases take place at birth [10] and rarely affect the first pregnancy [10]. Isoimmunization may lead to adverse maternal and foetal/neonatal outcomes such as hypertensive disease of the newborn (HDN) [11]. Mild jaundice, anaemia, and hydrops fetalis are the main signs of HDN. Extra medullary haematopoiesis (due to anaemia) may result in hepatosplenomegaly with resultant risks during labour and delivery, such as asphyxia and splenic rupture. In the post-natal period, the neonate may develop jaundice, anaemia, hypoglycaemia, difficulty in breathing, or death [11]. The laboratory findings vary with the severity of HDN and may include hyperbilirubinemia, reticulocytosis, elevated nucleated RBC count, thrombocytopenia, leukopenia, hypoalbuminemia, and RhD-ve blood type [12]. RhD-ve has been associated with the development of pre-eclampsia, placental abruption, cesarean delivery due to non-reassuring foetal status [13], oligohydramnios, and polyhydramnios [14].

Despite the availability of anti-D prophylaxis to prevent sensitization, gaps in its uptake, timing, and follow-up remain in many low- and middle-income countries. There is limited local data on the burden, risk factors, and pregnancy outcomes associated with Rh isoimmunization in Kenya, which hinders the formulation of effective management protocols and prevention strategies. This study aimed to determine the prevalence, sociodemographic characteristics, and pregnancy outcomes of Rhesus isoimmunized women managed at the Kenyatta National Hospital.

## Methods

**Study design and setting:** this cross-sectional study was conducted at Kenyatta National Hospital, a teaching and referral facility in East and Central Africa. The Department of Obstetrics and Gynaecology manages about 1,200 antenatal clients and 2,000 deliveries monthly.

**Study population:** the study included RhD-negative women attending ANC between January 2013 and December 2019. Of 216 records reviewed, 194 with complete data were analysed. Inclusion criteria were complete records of RhD-negative women with delivery details; files with missing data were excluded.

**Data collection:** data was extracted using a structured tool. At booking, all women undergo ABO and RhD typing. RhD-negative mothers receive anti-D immunoglobulin (300 μg) between 28 and 34 weeks and postpartum if the baby is RhD-positive. Information on demographics, clinical history, and outcomes was retrieved from hospital records.

**Definitions:** Rhesus isoimmunization was defined as the presence of maternal anti-D antibodies. Outcomes included anaemia, antepartum haemorrhage, hydrops fetalis, IUFD, birth weight <2500 g, APGAR <7, and Neonatal Intensive Care Unit (NICU) admission.

**Statistical analysis:** data were analysed using SPSS v23. Descriptive statistics summarized characteristics. Fisher´s exact test assessed associations between isoimmunization and outcomes. Significant variables (p<0.05) were entered into multivariable logistic regression to generate adjusted odds ratios (aORs), 95% CIs, and p values.

**Figure 1:**
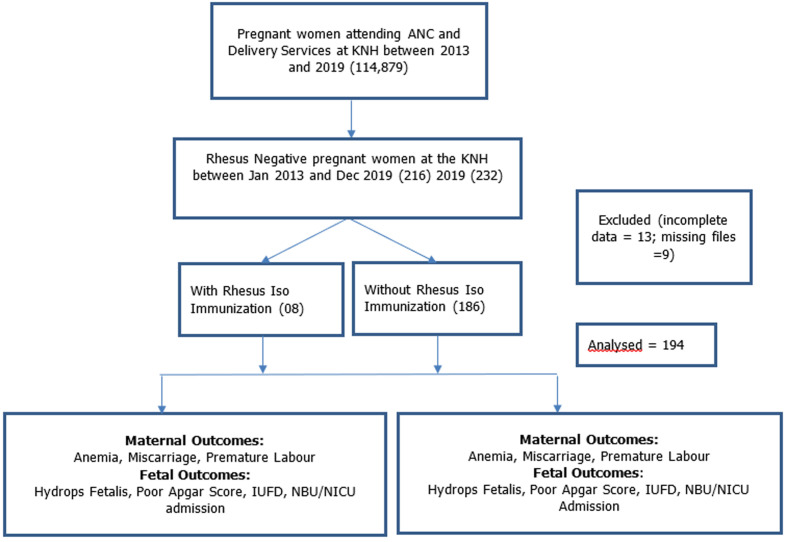
study flow diagram

**Ethical considerations:** ethical approval was obtained from the KNH-University of Nairobi (UoN) Ethics and Research Committee (Ref: KNH-ERC/A/124). Patient confidentiality was maintained, and identifiers were omitted (Figure 1).

## Results

**General characteristics of the study population:** a total of 194 mothers were assessed, of whom 8 (4.1%) had Rhesus isoimmunization. Most participants (53.6%) were aged 25-35 years, 27.8% were 19-24 years, and 18.6% were 36-49 years. Nearly half (49%) had attained secondary education, 38.7% tertiary, and 12.3% primary education. The majority were married (91.2%) and multigravida (69.1%) (Table 1).

**Table 1:** characteristics of the Rhesus-negative women who attended the antenatal clinic at the Kenyatta National Hospital between January 2013 and December 2019

Characteristic	N=194	Proportion (%)
Age; mean (SD)	28.64 (5.49)	
**Age**		
19 - 24	54	27.8
25 - 35	104	53.6
36 - 49	36	18.6
**Level of education**		
Primary	24	12.3
Secondary	95	49.0
Tertiary	75	38.7
**Marital status**		
Single	17	8.8
Married	177	91.2
**Parity**		
Primi-gravidae	60	30.9
Multi-gravidae	134	69.1

Regarding blood group, 41.8% were O-negative, 33.5% A-negative, 19.6% B-negative, and 2.1% AB-negative. Partner blood group was documented for 30 participants; most were B-positive (73%) (Table 2).

**Table 2:** blood group types for the Rhesus-negative and the partners who attended the antenatal clinic at the Kenyatta National Hospital

Variable	Frequency (n=194)	Proportion (%)
**Patient's blood group**		
A-ve	65	33.5
AB-ve	4	2.1
B-ve	38	19.6
O-ve	81	41.8
**Partners' blood group (n=30)**		
A+ve	6	3
B+ve	22	11
O+ve	2	1
Missing	164	85

**Maternal factors and isoimmunization:** among women with isoimmunization, 75% had experienced a prior pregnancy loss, with none documented as having received anti-D prophylaxis after the loss. Positive ICT titres were recorded in 87.5% of isoimmunized women. Isoimmunization was significantly associated with prior miscarriage (62.5% vs 16.7% in non-isoimmunized; p=0.006). Other maternal factors, including anemia (p=0.239), cesarean delivery (p=0.263), and antepartum hemorrhage (p=0.226), were not statistically associated with isoimmunization (Table 3).

**Table 3:** adverse maternal and foetal outcomes among Rhesus-negative women at the Kenyatta National Hospital, between January 2013 and December 2019

Variable	Isoimmunized	Not isoimmunized	P value
**Maternal outcomes**			
Anaemia (n=9)	1 (20)	9 (4.9)	0.239
Miscarriage (n=36)	5 (62.5)	31 (16.7)	0.006
Mode of delivery (CS) (n=133)	4 (50.0)	129 (69.4)	0.263
Antepartum haemorrhage (n=6)	1 (12.5)	5 (2.7)	0.226
**Fetal and neonatal**	**Outcomes**		
Hydrops fetalis	3 (37.5)	1 (0.5)	<0.001
IUFD	5 (62.5)	3 (1.6)	<0.001
Gestation at birth	6 (85.7)	10 (5.5)	<0.001
Fetal weight (<2500)	6 (85.7)	8 (4.3)	<0.001
Apgar score	6 (75.0)	4 (2.2)	<0.001
Admission to NICU	3 (37.5)	5(2.7)	0.002

IUFD: intrauterine fetal demise; NICU: neonatal intensive care unit

**Fetal and neonatal outcomes:** isoimmunization was strongly associated with adverse fetal and neonatal outcomes. Hydrops fetalis occurred in 37.5% of isoimmunized pregnancies versus 0.5% of non-isoimmunized (p<0.001), while intrauterine fetal demise (IUFD) was reported in 62.5% versus 1.6% (p<0.001). Isoimmunized women were more likely to deliver prematurely and have infants with birth weight <2500g (85.5% vs 4.3%), poor APGAR scores (75% vs 2.2%), and NICU admission (37.5% vs 2.7%), all p<0.001 except NICU (p=0.002).

## Discussion

This study aimed to determine the prevalence of Rhesus iso-immunization among Rh-negative pregnant women and assess associated maternal and fetal outcomes. Among the 194 women assessed, the prevalence of Rhesus isoimmunization was 4.1%, comparable to 5.5% reported by Adeyemi *et al*. in Ogbomosho, Nigeria, but lower than the 9.1% observed in Kaduna, Nigeria [15]. Routine antenatal prophylaxis has been reported to reduce the risk of sensitization to as low as 0.2% [2]. In our study, isoimmunized women were more likely to experience adverse maternal outcomes such as miscarriage (62.5% vs 16.7%, p=0.006), though differences in cesarean delivery, antepartum hemorrhage, and mild anemia were not statistically significant (p>0.05). These findings are consistent with previous studies showing that iso-immunization increases the risk of complications during pregnancy and postpartum [4,14,16].

Fetal complications were markedly higher among isoimmunized women. Hydrops fetalis occurred in 37.5% of isoimmunized pregnancies compared to 0.5% in the non-isoimmunized group. Similarly, intrauterine fetal demise was reported in 62.5% versus 1.6% (p<0.001). These rates exceed those reported in Bangladesh, where fetal death among iso-immunized women was 14% [17]. The elevated rates in our cohort reflect limited early fetal surveillance and a lack of intrauterine interventions for high-risk pregnancies.

Neonatal outcomes were also significantly worse among infants born to iso-immunized mothers. Low birth weight (<2500g) occurred in 85.5% versus 4.3% of non-isoimmunized pregnancies (p<0.001), poor APGAR scores in 75% versus 2.2% (p<0.001), and NICU admission in 37.5% versus 2.7% (p=0.002). Similar trends were reported in Nigeria, where 31.4% of neonates in the iso-immunized group had poor APGAR scores and required NICU admission [8]. The higher proportions in this study, partly explained by the small number of isoimmunized women, emphasize the need for targeted interventions to monitor and manage high-risk pregnancies among Rh-negative women.

The findings of this study have important clinical and public health implications. They show the need for routine antenatal anti-D prophylaxis, early identification of Rh-negative women, and enhanced fetal monitoring to prevent alloimmunization and reduce adverse outcomes. Moreover, our results highlight the importance of documenting partner blood group and prior pregnancy history, which are crucial for risk stratification and management planning.

The study is limited by its retrospective design, which is prone to gaps in documentation. Additionally, the low number of women with isoimmunization limited statistical power, and the use of Fisher´s exact test may be conservative, potentially underestimating associations. Consequently, we could not perform more robust correlation analyses between maternal and neonatal outcomes. Despite these limitations, the study provides valuable insights into maternal and fetal risks associated with Rhesus iso-immunization in this setting and supports the implementation of targeted prophylaxis and monitoring strategies.

## Conclusion

Rh-negative pregnancies represent an important subgroup of the obstetric population at KNH, with a prevalence of Rhesus isoimmunization of 4.1%. Although the number of isoimmunized women was low, they experienced significantly higher rates of adverse maternal and fetal outcomes, including miscarriages, hydrops fetalis, intrauterine fetal demise, low birth weight, poor APGAR scores, and increased NICU admissions compared to non-isoimmunized women. These findings highlight the clinical importance of early identification of Rh-negative women, routine antenatal anti-D prophylaxis, and enhanced fetal surveillance to reduce sensitization and improve maternal and neonatal outcomes in this high-risk population.

### What is known about this topic

- Rhesus isoimmunization occurs in Rh-negative pregnancies and can cause maternal and fetal complications;
- Routine antenatal anti-D prophylaxis significantly reduces sensitization during pregnancy;
- Isoimmunization is associated with adverse outcomes such as hydrops fetalis, fetal demise, and poor neonatal health indicators.

### What this study adds

- Prevalence of Rhesus isoimmunization at KNH was 4.1% among Rh-negative women;
- Isoimmunized women had significantly higher rates of miscarriage, hydrops fetalis, and IUFD;
- Infants born to isoimmunized mothers were more likely to have low birth weight, poor APGAR scores, and require NICU admission.
